# Preparation of a Molecularly Imprinted Polymer on Polyethylene Terephthalate Platform Using Reversible Addition-Fragmentation Chain Transfer Polymerization for Tartrazine Analysis via Smartphone

**DOI:** 10.3390/polym16101325

**Published:** 2024-05-08

**Authors:** Christian Jacinto Hernández, Raúl Medina, Ily Maza Mejía, Mario Hurtado, Sabir Khan, Gino Picasso, Rosario López, María D. P. T. Sotomayor

**Affiliations:** 1 Laboratory of Instrumental Analysis Environment, Faculty of Sciences, National University of Engineering, Av. Tupac Amaru 210, Rimac 15333, Lima, Peru; christian@uni.edu.pe (C.J.H.); raul.medina.r@uni.pe (R.M.);; 2 Technology of Materials for Environmental Remediation (TecMARA) Research Group, Faculty of Sciences, National University of Engineering, Av. Tupac Amaru 210, Rimac 15333, Lima, Peru; gpicasso@uni.edu.pe; 3 Facultad de Ingeniería de Petróleo, Gas Natural y Petroquímica, Universidad Nacional de Ingeniería, Av. Tupac Amaru 210, Rimac 15333, Lima, Peru; mhurtadoc@uni.edu.pe; 4 Department of Exact Sciences and Technology, State University of Santa Cruz, Ilhéus 45662-900, BA, Brazil; skhan@uesc.br; 5 Institute of Chemistry, State University of São Paulo (UNESP), Araraquara 14801-970, SP, Brazil; 6 National Institute for Alternative Technologies of Detection, Toxicological Evaluation and Removal of Micropollutantans Radioactives (INCT-DATREM), Araraquara 14801-970, SP, Brazil

**Keywords:** tartrazine, PET, RAFT polymerization, smartphone, digital image colorimetry (DIC)

## Abstract

This work describes the preparation of a molecularly imprinted polymer (MIP) platform on polyethylene terephthalate (MIP-PET) via RAFT polymerization for analyzing tartrazine using a smartphone. The MIP-PET platform was characterized using Fourier transform infrared (FTIR) techniques, Raman Spectroscopy, X-ray photoelectron spectroscopy (XPS), and confocal microscopy. The optimal pH and adsorption time conditions were determined. The adsorption capacity of the MIP-PET plates with RAFT treatment (0.057 mg cm^−2^) was higher than that of the untreated plates (0.028 mg cm^−2^). The kinetic study revealed a pseudo-first-order model with intraparticle diffusion, while the isotherm study indicated a fit for the Freundlich model. Additionally, the MIP-PET demonstrated durability by maintaining its adsorption capacity over five cycles of reuse without significant loss. To quantify tartrazine, images were captured using a smartphone, and the RGB values were obtained using the ImageJ^®^ free program. A partial least squares regression (PLS) was performed, obtaining a linear range of 0 to 7 mg L^−1^ of tartrazine. The accuracy of the method was 99.4% (4.97 ± 0.74 mg L^−1^) for 10 samples of 5 mg L^−1^. The concentration of tartrazine was determined in two local soft drinks (14.1 mg L^−1^ and 16.5 mg L^−1^), with results comparable to the UV–visible spectrophotometric method.

## 1. Introduction

Tartrazine is a yellow dye used in beverages, juices, sweets, and various food products. However, some studies suggest that its use may lead to health issues such as headaches, neurotoxicity, genotoxicity, and carcinogenicity [1]. The daily intake of tartrazine in humans ranges from 0 to 7.5 mg kg^−1^ of body weight (reported by the Joint FAO/WHO Expert Committee on Food Additives). The mutagenic, carcinogenic, and toxic effects of azo dyes like tartrazine may result from the direct effect of the dye on the reductive biotransformation of the azo bond during its metabolism [2].

Multiple techniques have been developed for the determination of tartrazine, such as spectrophotometry [3], thin-layer chromatography, voltammetry [4], and high-precision liquid chromatography [5,6]. A summary of other techniques for the determination of tartrazine in food can be found in Rovina’s work [7]. These techniques are effective but have the drawback of being expensive, employing complicated sample preparation procedures, as well as requiring extended analysis time and large amounts of toxic solvents.

Molecularly imprinted polymers (MIPs) are synthetic analogs of natural antibody and antigen biological systems. As such, they operate as a “lock and key” mechanism to selectively bind the analyte with which they were imprinted during synthesis. MIPs offer the potential specificity and selectivity of biological receptors with the explicit advantages of durability under environmental conditions and low cost [8]. To achieve high binding capacity and efficiency, MIPs must possess a homogeneous distribution of recognition cavities [9].

The selected polymerization method plays a crucial role in providing uniformity to the MIP structure. Conventional free radical polymerization (FRP) methods result in a heterogeneous distribution of binding sites and low affinity and imprinting efficiency, as well as irregular internal morphology and porosity in the MIP structure [10]. Instead, reversible deactivation radical polymerization (RDRP) techniques increase structural uniformity and enhance binding properties [11]. Among these techniques is reversible addition-fragmentation chain transfer (RAFT) polymerization, which has been widely used for the preparation of tailored MIPs under controlled conditions, resulting in polymeric structures with more uniform cavities than conventional techniques [12].

Image analysis applied to chemical analysis is quite recent. Digital images of samples, if processed correctly, can contain valuable information about their composition. Color evaluation is highly beneficial in digital image analysis [13]. The use of smartphones combined with digital image colorimetry (DIC) sensors or devices can be used as detectors or quantifiers of colored substances, as the digital images from smartphone cameras, combined with suitable image processing applications and software, can establish the relationship between color intensity and the quantity of the colored substance. To establish the correlation between the digital image signal and the analyte concentration, two steps will be undertaken. Firstly, color quantification will be performed using image analysis software such as Adobe Photoshop CC (v.21), Matlab 9.10, ImageJ (1.53k 6 July 2021), and Color Pilot 4.51, customized by manufacturers. The second step involves studying the correlation between the chromatic value or its function and the analyte concentration through appropriate mathematical treatment. Within a specific concentration range, the chromaticity of the colored substance is related linearly or nonlinearly to its concentration, enabling the analysis of the sample after establishing a calibration curve with the original color values [14] using chemometric methods [15] or machine learning techniques [16]. Overall, these applications using smartphones can simplify traditional analysis methods, resulting in fast and user-friendly technologies [17]. This research introduces an innovative material for conducting fast, cost-effective, and user-friendly analyses utilizing molecularly imprinted polymers. The purpose of using the smartphone is not to replace the chemical information provided by other instrumental techniques, such as spectroscopic techniques, but rather to take advantage of the fact that a high-resolution photograph taken with a smartphone camera is more economical and convenient. The wide availability of digital images offers current opportunities for the development of rapid and low-cost digital image analysis techniques on smartphones for both qualitative and quantitative analyses.

The aim of this study is to develop a platform based on MIP and mediated by RAFT for digital image colorimetry (DIC) using smartphones, with the capability to quantify tartrazine in carbonated beverages. An MIP-PET device mediated with RAFT reagent will be obtained, representing an innovation in tartrazine adsorption. Additionally, a DIC methodology using smartphones will be developed through a chemometric calibration approach, thereby establishing the foundation for the application of analyzing other substances generating color in the MIP, besides applying other mathematical and/or artificial intelligence methods.

## 2. Experimental Section

### 2.1. Chemical and Solutions

All reagents used were of analytical grade. Solutions were prepared with ultrapure water (18 MΩ at 25 °C). Tartrazine (TZ), *N*,*N*′-methylenebisacrylamide (NMBA), acrylamide (AA), potassium persulfate (KPS), hydrogen peroxide, and benzophenone were purchased from Sigma-Aldrich (St. Louis, MO, USA). Cumyl dithiobenzoate was used as a RAFT reagent and purchased from Sigma-Aldrich. 1,4-dioxane was purchased from Supelco, and ammonia solution (23–28%) and hydrochloric acid (33%) were purchased from Merck. The dyes used in the selectivity study, Basic Red 46, Methyl Green, Sunset Yellow, and Yellow HE_3_G, were acquired from Sigma-Aldrich.

### 2.2. Preparation of the MIP-PET Platform

#### 2.2.1. Activation of PET Plates

In the first step, PET plates were activated following the methodology of Kaymaz [18] of PET plates were obtained and cut into 2 cm × 2 cm × 0.6 cm sheets. Subsequently, the plates were washed with a methanol/water (50:50) mixture for 30 min and air-dried. In the hydrolysis stage, the plates were immersed in 2 mol L^−1^ NaOH at 70 °C for 90 min, then submerged in a solution of acetic acid 50% at 70 °C for 15 min. Following this, the plates were washed with distilled water and dried in a vacuum. The PET plates underwent an oxidation process being immersed in 0.6 mol L^−1^ H_2_O_2_ for 3 h under UV radiation (LED 365 nm, 75 W) at 10 cm from the plates. The solution was adjusted to pH 3 with 0.1 mol L^−1^ HCl.

Next, the PET plates were submerged in a 10% benzophenone solution in dioxane, subjected to ultrasound for 10 min, and then UV radiation for 60 min at 10 cm distance from the PET plates. Afterward, the plates were removed, immersed in dioxane for 5 min, and immediately transferred to the reaction flask for polymerization.

#### 2.2.2. Polymerization of MIP on PET Plates (MIP-PET)

A procedure adapted from a previously documented MIP synthesis [15] was employed. The polymerization process began by mixing 0.1 mmol of TZ with 0.2 mmol of AA in 40 mL of water and stirring for 2 h. Subsequently, nitrogen bubbled for 10 min, followed by the addition of 10 mmol of *N*,*N*′-methylenebisacrylamide, 1 mL of the RAFT reagent dissolved in ethanol (2 mg mL^−1^), and 1 mL of potassium persulfate in water (1 mg mL^−1^). Nitrogen bubbling was repeated for 10 min, the PET plates treated with benzophenone were added, and the reaction flask sealed the nitrogen atmosphere. The mixture was stirred and heated to 70 °C, allowing polymerization to proceed for 3 h.

In addition, the reaction flask was uncovered and allowed to cool to room temperature. The MIP-PET was washed with distilled water and placed in a flask with a 20% ammonia washing solution, subjected to ultrasound for 10 min at 15 °C. The solution was then changed, and the washing process with 20% ammonia continued under agitation at 200 rpm at room temperature for 24 h. Finally, the samples were washed with distilled water and dried under vacuum. Finally, the control material, NIP-PET plates, was prepared following the same procedure but without the addition of tartrazine.

#### 2.2.3. Instrumentation

The absorbance measurements of tartrazine solutions were performed using a Shimadzu (Kyoto, Japan) UV-1800 UV–visible spectrophotometer. Spectra were obtained in the range of 200 to 600 nm.

Infrared spectra were obtained in attenuated total reflectance (ATR) mode using a Shimadzu^®^ model IRPrestige 21 FTIR spectrophotometer in the range of 400 to 4000 cm^−1^. Spectra were acquired through 20 scans with a resolution of 4 cm^−1^.

Chemical analysis of the surface of the PET samples was carried out using X-ray photoelectron spectroscopy (XPS) with a conventional XPS spectrometer (ScientaOmicron ESCA+) equipped with a high-performance hemispherical analyzer (EAC2000) with 128 channels and monochromatic Al Kα radiation (hν = 1486.6 eV) as the excitation source. The operating pressure in the ultra-high vacuum (UHV) chamber during the analysis was 10^–9^ Pa. High-resolution XPS spectra were recorded at a constant pass energy of 30 eV with 0.05 eV per step. An electron flood gun (CN10) was used as a charge neutralizer.

To evaluate the surface roughness during PET treatment and the thickness of the MIP deposited on the PET, a confocal laser scanning microscope (LEXT OLS 4000) controlled by Olympus 1.3.5 software was used. Scanning electron microscopy (SEM) images were recorded using a Zeiss EVO MA 10 (Dublin, CA, USA).

The surface wettability of clean, oxidized, and benzophenone-immobilized PET surfaces was measured at room temperature using an optical contact angle apparatus (Dataphysics, Germany) with an attached camera and SCA20.2.0 software. Images between the substrate and the distilled water droplet were obtained using 7 μL of water.

#### 2.2.4. Adsorption Test

The MIP-PET plates, cut into 1 cm × 1 cm pieces, were placed in 10 mL of tartrazine solution at pH 3.0 at the fixed concentration. The system was shaken for up to 2 h on an orbital shaker at 200 rpm, and the absorbance of the final solution was measured at 427 nm. The amount of adsorbed tartrazine was determined using a calibration curve. The removal percentage was calculated according to Equation (1):

(1) $$Removal\;\left(\%\right)=\frac{\left(C_{o}-C_{e}\right)}{C_{e}}\times 100$$

The adsorption capacity *Q* was calculated using Equation (2):

(2) $$Q\left(\frac{mg}{cm^{2}}\right)=\left(C_{o}-C_{e}\right)V$$

where *Q* (mg cm^−2^) is the experimental adsorption amount on the 1 cm × 1 cm plate, *C_o_* (mg L^−1^) is the initial analyte concentration, *Ce* (mg L^−1^) is the analyte concentration at equilibrium, and *V* is the volume of the solution in liters.

#### 2.2.5. Image Capture with RadesPhone Device

RadesPhone was used, a device previously reported by our research group [15], for image capture. Moreover, 1 cm^2^ MIP–PET or NIP–PET plates were submerged in a tartrazine solution at pH 3.0 and agitated on an orbital shaker for 2 h. Subsequently, the plates were washed and vacuum-dried. For image capture, the plates were placed on the RadesPhone and a Samsung S20 FE smartphone model SM–G780F, with a 12 MP rear camera, was positioned 15 cm from the plate and at a 90° angle to the surface normal. Images were recorded in JPG format with a resolution of 1816 × 4032 pixels.

#### 2.2.6. Digital Image Colorimetry

The color intensity measurement on the MIP-PET plates exposed to the tartrazine solutions is performed by extracting the RGB color values from the images at a size of 16 × 16 pixels using ImageJ 1.53k software. These colors were then converted into other color channels, such as HSV and CMYK, using Equations (3) and (4) [19], which have been reported for use in other studies [13,15].

(3) $$\left.\begin{array}{l} C=1-\frac{R}{255}\\ M=1-\frac{G}{255}\\ Y=1-\frac{B}{255}\\ K=\min(C,M,Y) \end{array}\right\}$$

(4) $$\left.\begin{array}{c} V=MAX(R,G,B)\\ S=\left\{\begin{array}{c} 0\;if\;V=0\\ 1-\frac{MIN}{V}otherwise \end{array}\right\}\\ undefined,\;if\;MAX=MIN\\ H=\left\{\begin{array}{l} 60{}^{\circ}\times \frac{G-B}{MAX-MIN}+0{}^{\circ},if\;MAX-R\;and\;G\geq B\\ 60{}^{\circ}\times \frac{G-B}{MAX-MIN}+360{}^{\circ},\;if\;MAX-R\;and\;G<B\\ 60{}^{\circ}\times \frac{B-R}{MAX-MIN}+120{}^{\circ},if\;MAX=G\\ 60{}^{\circ}\times \frac{R-G}{MAX-MIN}+240{}^{\circ},if\;MAX=B \end{array}\right. \end{array}\right\}$$

The data matrix of RGB, CMYK, and HSV color channels were correlated with the concentrations of tartrazine exposed to the MIP-PET plates. The color values were standardized, and a multivariate PLS calibration was performed, evaluating the range of working tartrazine concentrations, the limit of quantification, and the limit of detection.

## 3. Results and Discussion

### 3.1. Synthesis of MIP-PET and NIP-PET

The process of obtaining MIP-PET involved adapting the procedure developed by Kaymaz et al. [18] to obtain tartrazine MIP films on PET plates. This process consists of four stages: hydrolysis, oxidation, benzophenone immobilization, and polymerization.

**Figure 1:**
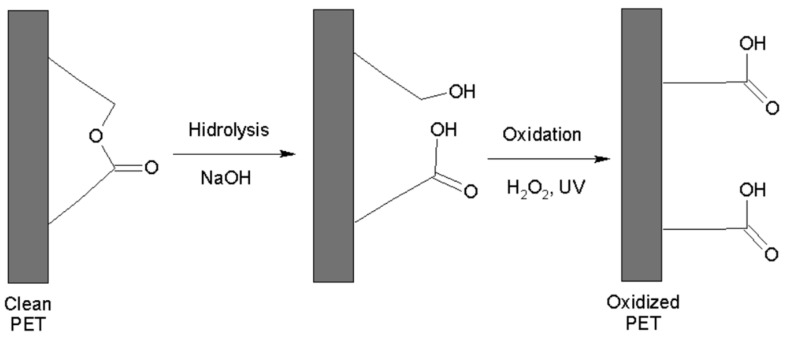
Hydrolysis procedure and oxidation of PET.

The polymer grafting process onto PET occurs when the latter is treated with benzophenone [20]. Korolkov reported that oxidized surfaces of PET containing carboxyl groups adsorb a higher amount of benzophenone compared to non-oxidized surfaces. The proposed reaction for hydrolysis and oxidation is shown in Figure 1 [21].

**Figure 2:**
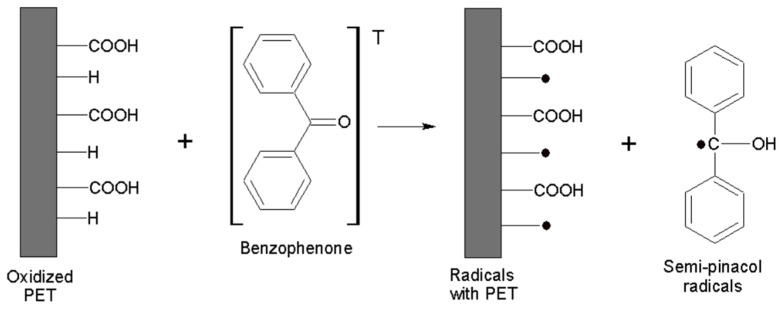
Radical Formations on PET surface.

The benzophenone adsorbed by the carboxyl groups of the oxidized PET plate and exposed to UV radiation reaches a triplet state, removing a hydrogen atom from the C–H groups of PET, forming radicals on the surface (Figure 2) [22,23].

**Figure 3:**
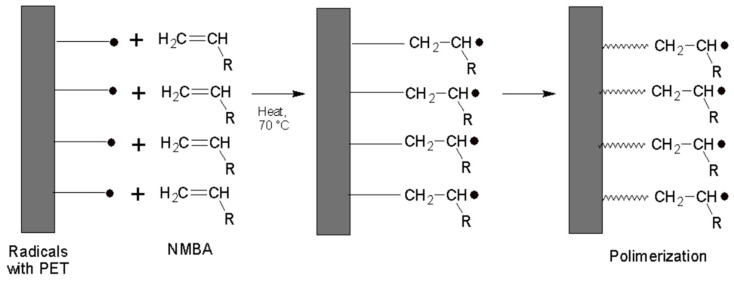
Polymerization of the PET plate.

The semi-pinacol radicals formed after the scission of the benzophenone attached to the PET surface are not prone to initiate polymerization [22]; thus, KPS is crucial for the polymerization initiation, along with the CDB for the RAFT process. The graft polymerization process occurs with the radicals of PET (Figure 3).

### 3.2. Optimization of the Synthesis

In this study, an investigation and optimization of various parameters involved in the synthesis of MIP-PET were conducted with the aim of maximizing the amount of adsorbed tartrazine. The quantity of adsorbed tartrazine was quantified by measuring the absorbance of solutions at a wavelength of 427 nm. The parameters evaluated in the preparation of MIP-PET included the molar ratio between the monomers and the RAFT reagent, the ratio between the potassium persulfate as initiator and the cumyl dithiobenzoate RAFT reagent, and the conditions for washing the MIP.

The initial MIP-PET plates were prepared with a TZ:AA:NMBA molar ratio of 1:2:100. This selection was made with the aim of obtaining an MIP with low residual coloration after washing, a prerequisite for subsequent color analysis tests with the smartphone. Initial adsorption tests of MIP-PET were carried out with tartrazine solutions at pH 3 and a contact time of 1 h. The quantity of adsorbed tartrazine was determined spectrophotometrically at 427 nm, using the equation to calculate Q.

Different solvents and conditions for the removal of tartrazine from MIP-PET were investigated. The use of water at room temperature and at 70 °C, as reported by Arabzadeh et al. [23], was not suitable for our work, as the MIP retained a yellow color and showed low adsorption capacity. Tartrazine extraction with 20% ammonia has been employed in previous research [15,24]. This method was selected as the most suitable, using a washing protocol consisting of 15 min of treatment with 20% ammonia solution under ultrasound and then an additional 24 h of agitation.

#### 3.2.1. Effect of the Oxidation Time

**Figure 4:**
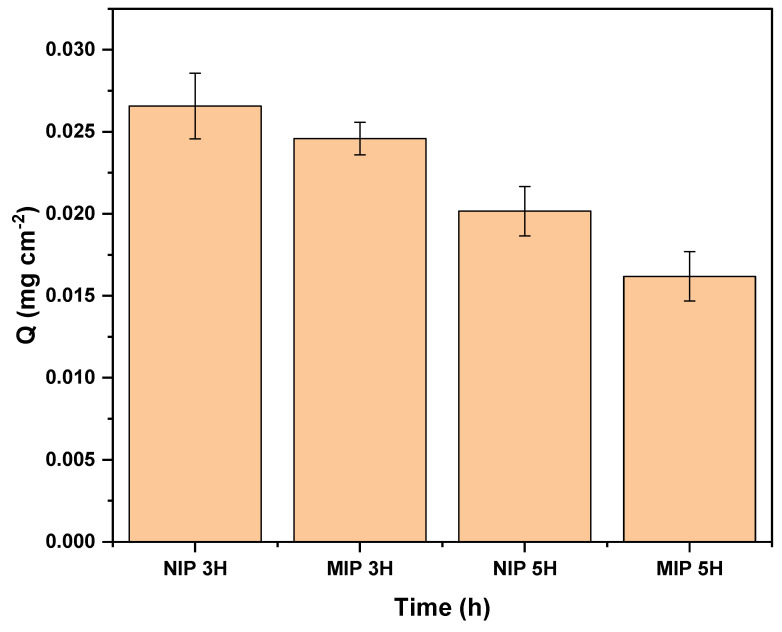
Adsorption capacity of MIP-PET and NIP-PET prepared with oxidation times of 3 h (NIP 3H and MIP 3H) and 5 h (NIP 5H and MIP 5H). Plates with a molar ratio of TZ:AA:NMBA of 1:2:100, without RAFT reagent, adsorption with 1 cm^2^ plate, 10 mL of 10 mg L^−1^ tartrazine at pH 3.

The PET plates undergo three preliminary steps before the MIP grafting process. One of these steps is the oxidation of PET with hydrogen peroxide (H_2_O_2_) and ultraviolet light. This step is crucial for the subsequent immobilization of benzophenone, as reported by Korolkov et al. [21]. An analysis of the oxidation time was conducted to adapt the procedure to the UV lamp equipment available in our laboratory. Figure 4 illustrates the results of the influence of oxidation time on the adsorption capacity of MIP-PET.

Kaymaz [18] mentions a 5 h oxidation time in their study. In this work, we also evaluated an oxidation time of 3 h. As observed in Figure 4, the MIP-PET plates prepared with a 3 h oxidation time exhibit a higher adsorption capacity (Q) than the plates oxidized for 5 h. The discrepancy with Kaymaz’s findings could be attributed to the difference in the intensity of the UV light source used in each study. Kaymaz does not specify the type of lamp used, whereas in our case, we used an LED lamp with 365 nm and 75 W. Higher UV light intensity may degrade the surface of the PET, which could affect the efficiency of polymer fixation on the plate in the long term.

Figure 4 also shows that the NIP-PET absorbs more than the MIP-PET. This is because the RAFT reagent is not used in the preparation of NIP-PET.

#### 3.2.2. Molar Ratio of Monomers

As mentioned earlier, the TZ:AC:NMBA molar ratio of 1:2:100 was used because it presented a less intense tartrazine hue after washing, possibly due to the higher amount of NMBA in the formulation. The ratio between the sum of monomers (AA + NMBA) and the RAFT reagent influenced the degree of polymerization. Table 1 shows the Q values for the various proportions of monomers and RAFT reagents.

Table 1 shows that the MIP–PET–1 ratio exhibits the highest adsorption capacity, and in subsequent syntheses, this proportion was used. In the synthesis process of MIP mediated by the RAFT reagent, there are equilibrium stages that reveal the influence of the initial quantities of monomers (acrylamide and *N*,*N*′-methylene–bis-acrylamide), the radical initiator (KPS), and the RAFT reagent itself on obtaining a more homogeneous surface of the MIP. According to Abdollahi et al. [25], the monomer conversion rate is subject to the initiator concentration, the resulting polymerization degree from the sum of the monomers and the RAFT reagent, as well as the dispersion of the polymerization reaction rate.

#### 3.2.3. Molar Ratio of RAFT/KPS

**Figure 5:**
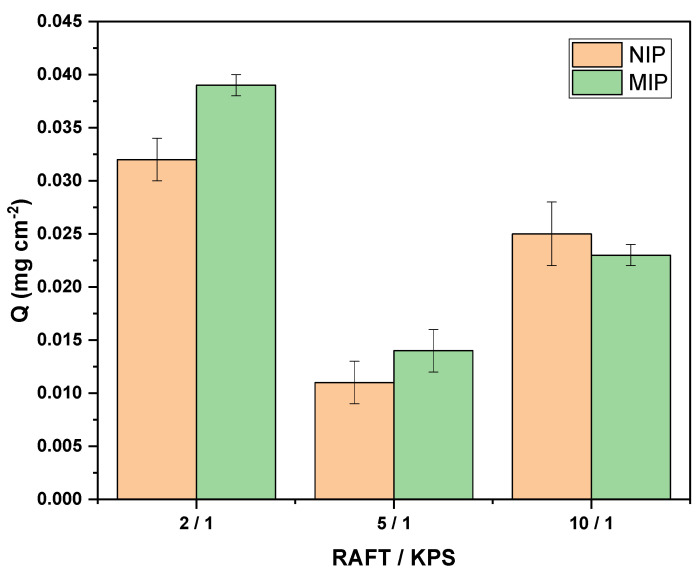
Values of Q for the different MIP-PET according to the molar ratio of RAFT/KPS. Adsorption of 1 cm^2^ plate, tartrazine concentration 10 mg L^−1^, and pH 3.

The RAFT/KPS ratio influences the degree of polymerization, which, in turn, determines the homogeneity of the MIP surface. Three RAFT/KPS ratios were evaluated: 2/1, 5/1, and 10/1 while maintaining the quantity of RAFT determined in the previous step. As observed in Figure 5, the MIP-PET prepared with a 2/1 molar ratio of RAFT/KPS = 2 exhibited the highest adsorption capacity. Furthermore, this sample of MIP-PET also shows the greatest difference in adsorption between the MIP and the NIP. Therefore, the RAFT/KPS ratio of 2/1 was used in all subsequent syntheses.

The molar ratio of RAFT to KPS is crucial for optimal control over the growth of polymer chains and, thus, the homogeneity of the MIP surface [26]. The initiator initiates polymerization of the monomers, while the RAFT agent controls the growth of the polymer chain through the reversibility of the reactions it participates in.

#### 3.2.4. Effect of the RAFT Treatment

In this study, the impact of treatment with the RAFT agent on the adsorption capacity of an MIP-PET for tartrazine was evaluated. MIP-PET plates were prepared with and without RAFT during the polymerization stage.

Adsorption tests with 10 mg cm^−2^ tartrazine at pH 3 showed a higher adsorption capacity (Q) in the plates with RAFT (Q = 0.057 ± 0.004 mg cm^−2^) compared to those without RAFT (Q = 0.028 ± 0.003 mg cm^−2^). This result is attributed to the control provided by RAFT-mediated polymerization over the polymer architecture, which facilitates tartrazine access to binding sites [27].

### 3.3. Characterization of PET and MIP-PET

The instrumental techniques employed in this study will provide information regarding the modification of PET during hydrolysis and oxidation treatments. To achieve this, measurements of contact angle and XPS spectroscopy will be utilized. To confirm the formation of MIP on the PET surface, techniques such as FTIR spectroscopy, confocal microscopy, SEM, and XPS spectroscopy will be employed. By employing these instrumental techniques for the characterization of MIP-PET plates, the adsorption of tartrazine onto this device can be elucidated, which, in turn, will be related to the amount of color captured by the smartphone camera, enabling digital image colorimetry analysis (DIC).

**Figure 6:**
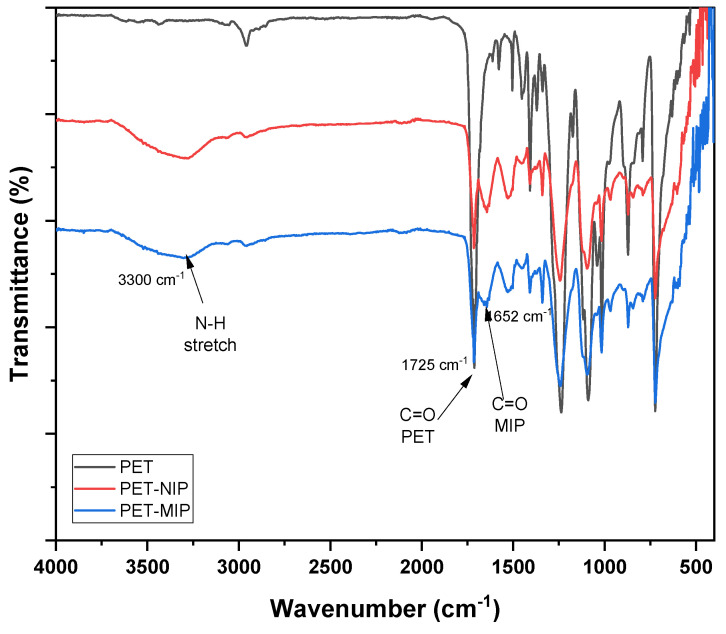
ATR–FTIR spectra of clean PET (PET) and plates grafted with NIP (PET–NIP) and MIP (MIP–PET).

The ATR-FTIR spectra of clean PET and MIP grafted onto the PET surface are presented in Figure 6. In the graph, the formation of both MIP and NIP on the PET surface can be observed due to the presence of two characteristic peaks of the MIP. At 3300 cm^−1^, the vibration peak corresponding to the NH_2_ group is observed, while at 1652 cm^−1^, the vibration peak related to the C=O bond is highlighted, both corresponding to the functional groups of the crosslinker *N*,*N*′-methylenebisacrylamide. Additionally, in all three spectra, the peak at 1725 cm^−1^ corresponding to the C=O bond present in the PET ester is identified. Infrared radiation can penetrate a few micrometers in the case of polymers, allowing for the detection of this peak in the MIP. 

**Figure 7:**
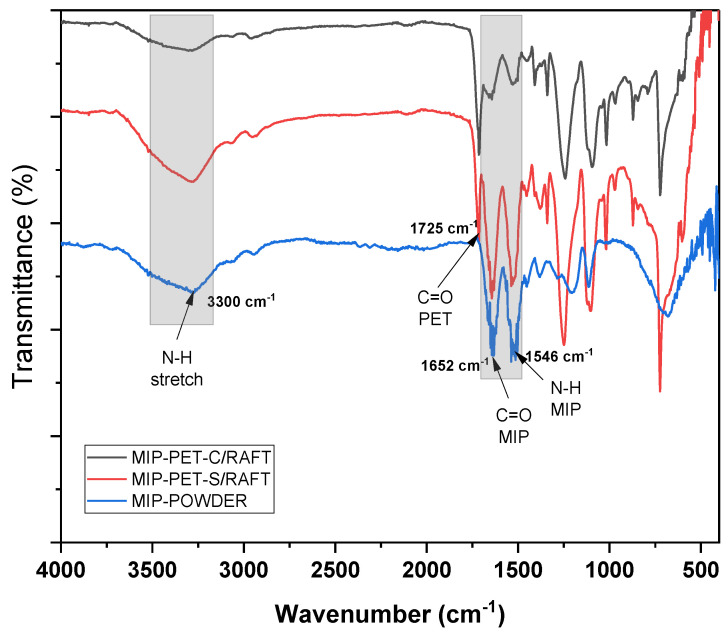
ATR–FTIR spectra of clean PET (PET) and plates grafted with RAFT–mediated MIP (MIP–PET–C/RAFT) and with MIP without RAFT (MIP–PET–S/RAFT) and MIP in powder (MIP–POWDER).

Figure 7 shows the ATR–FTIR spectrum of the MIP grafted onto the PET plate with and without RAFT mediation (MIP–PET C/RAFT and MIP–PET S/RAFT), along with the MIP obtained by precipitation in powder form (MIP–POWDER) [15]. All three spectra exhibit a peak at 3300 cm^−1^ attributed to the N–H stretching vibration, confirming the formation of the MIP. At 1725 cm^−1^, the C=O peak of PET is observed in the MIP–PET samples but not in MIP–POWDER, indicating the formation of MIP on PET. The PET peaks are attributed to the penetration of IR radiation into the MIP surface. Several peaks with different intensities are observed between MIP–PET C/RAFT and MIP–PET S/RAFT, suggesting differences in the thickness of the MIP formed on PET [14]. MIP–PET C/RAFT exhibits peaks with lower intensities, such as the band at 3300 cm^−1^ corresponding to the N–H peak, as well as peaks at 1652 cm^−1^ and 1546 cm^−1^ corresponding to C=O and N–H, respectively.

**Figure 8:**
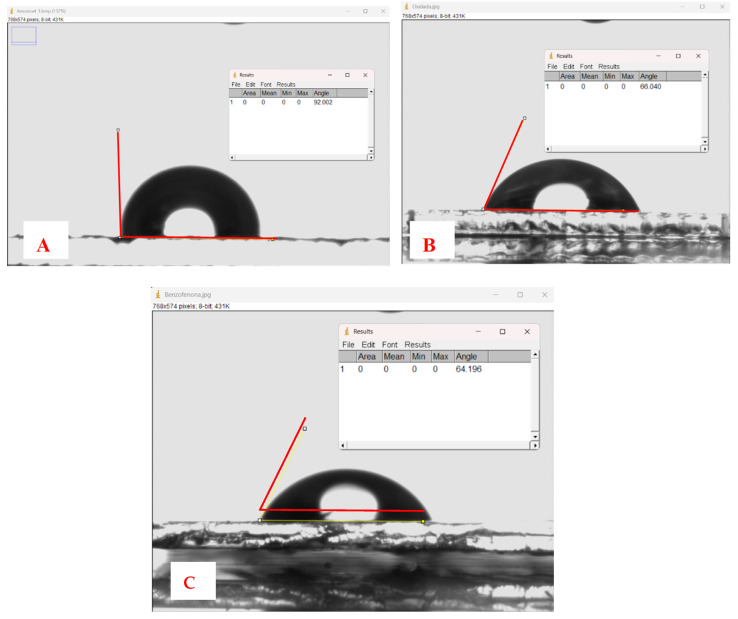
Images of water droplets and measurement of contact angles on (**A**) clean PET, (**B**) oxidized PET, and (**C**) PET with benzophenone.

The measurement of the contact angle on the PET surface at each treatment stage provides information about the surface changes induced by chemical treatment. These changes affect the wettability properties and the interaction between the PET surface and the liquids with which it comes into contact. Figure 8 shows the variation of the contact angles of the PET plates at different treatment stages. Figure 8A shows a contact angle of 92° for the clean PET plate, indicating low hydrophilicity. The oxidation process of PET transforms the ester bonds of the main chain into new free hydroxyl and carboxyl groups on the polymer surface (see Figure 1). This increases the hydrophilicity of the PET substrate and reduces the contact angle to 66° (Figure 8B). The immobilization of benzophenone slightly decreases the angle to 64° (Figure 8C), indicating a slight modification of the surface hydrophilicity.

The measurement of the contact angle in the pretreatment of PET plates is crucial, as benzophenone will be immobilized on the PET to initiate graft polymerization. The greater the number of carboxyl groups present on the surface of PET, the higher the adsorption capacity of benzophenone and, therefore, the capacity to graft MIP onto PET [28]. The hydrophilicity of the PET surface is inversely related to the contact angle. A lower contact angle indicates higher hydrophilicity, meaning that the liquid tends to wet the surface of the PET more easily [29].

Confocal scanning microscopy is a non–invasive and non–destructive technique used to obtain 3D topographic images of PET during its treatment, as well as the obtained MIP–PET and NIP–PET. Initially, an objective lens with a 5× magnification was selected, and for the analyses, a resolution magnification of 100× was used. Images were collected from six different points of each sample, and from these, the roughness and mean thickness parameters of our materials were calculated.

Morphological changes on the surface of PET due to oxidation and MIP formation are of particular interest, as they demonstrate surface functionalization occurrence. One of the parameters obtained from the confocal scanning microscope is the roughness value (Ra), which is a fundamental measure to quantify surface texture [29]. This value provides information about the height and frequency of irregularities present on the sample surface, allowing us to accurately characterize its topography.

**Figure 9:**
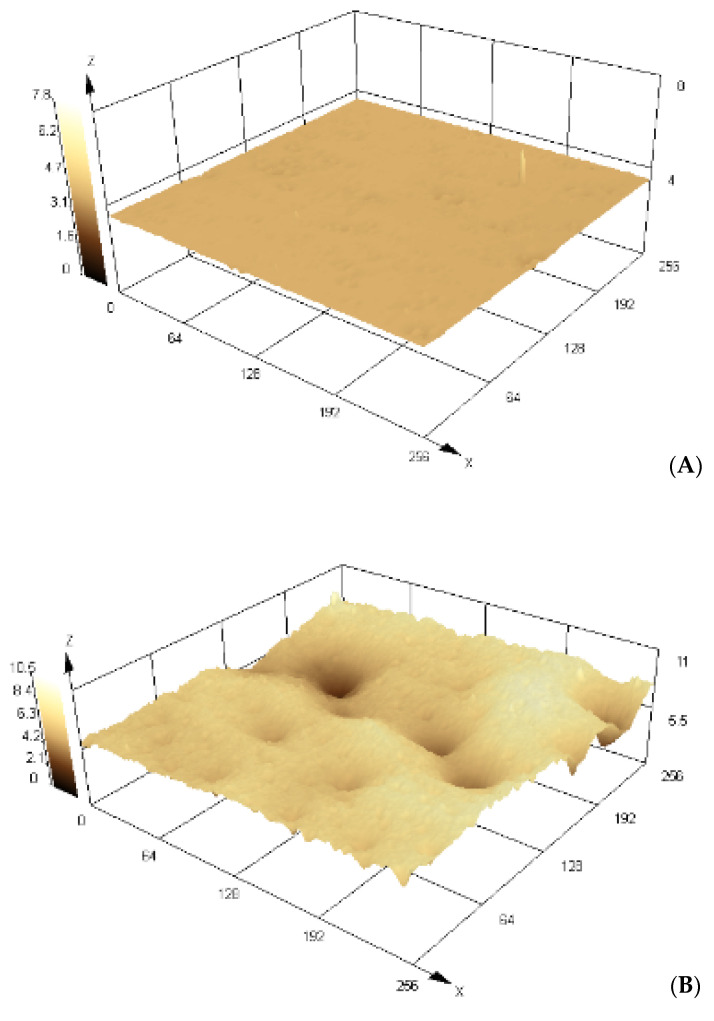

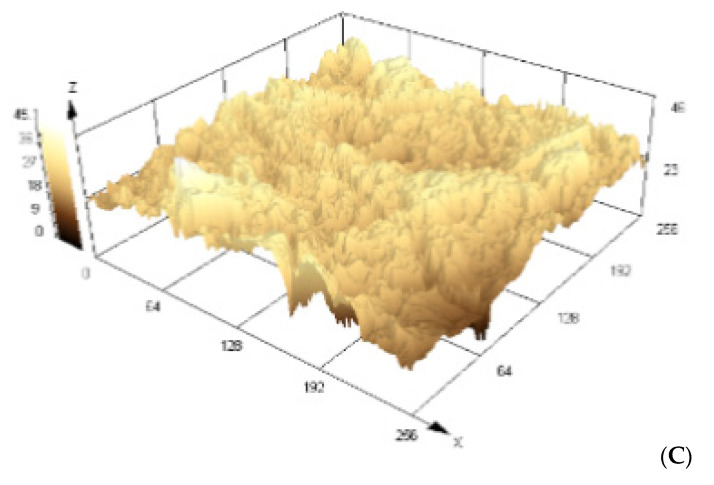
Images in 3D, obtained from a confocal scanning microscope of (**A**) clean PET, (**B**) oxidized PET, and (**C**) PET with benzophenone. Scale units in μm.

Figure 9 displays 3D images of PET plates during treatment before MIP grafting onto them. The image of the clean PET plate (Figure 9A), without treatment, shows a roughness value Ra of 0.017 μm (±0.004); after treatment with NaOH and H_2_O_2_, the oxidized PET plate (Figure 9B) shows a roughness value Ra of 0.258 μm (±0.050). This increase in roughness is due to PET etching in the UV/H_2_O_2_ medium. According to Korolkov [21], PET oxidation with H_2_O_2_/UV produces less surface damage and, therefore, a smoother surface than treatment with other oxidants. Treatment with benzophenone (Figure 9C) increases surface roughness with Ra = 1.594 μm (±0.30), which impacts the roughness of the obtained MIP–PET.

**Figure 10:**
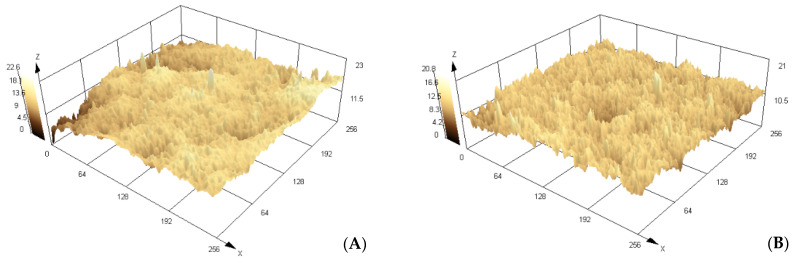

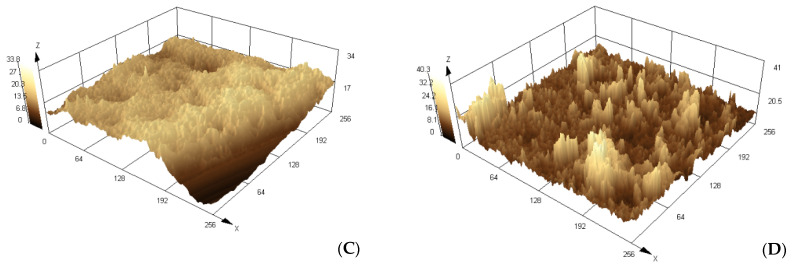
Images in 3D, obtained from a confocal scanning microscope of (**A**) NIP–PET (NIP), (**B**) MIP–PET 2/1 (MIP 2/1), (**C**) MIP–PET 5/1 (MIP 5/1), and (**D**) MIP–PET with tartrazine (MIP–T). Scale units in μm.

Figure 10 depicts 3D images of the surface of MIP supported on PET. The effect of the RAFT/KPS ratio on the surface characteristics of MIP has been evaluated. MIP–PET 2/1 (Figure 10B) and MIP–PET 5/1 (Figure 10C) have RAFT/KPS ratios of 2/1 and 5/1, respectively. It is observed that the MIP–PET with a higher RAFT proportion (MIP–PET 5/1) exhibits greater surface roughness (Ra = 1.052 μm) compared to MIP–PET 2/1 (Ra = 0.852 μm). This could generate greater heterogeneity and lower availability of active sites on the MIP surface. Additionally, the thickness of the MIP–PET 5/1 layer is greater (7.0 mm) than that of MIP–PET 2/1 (6.32 μm). Figure 10D shows the MIP–PET with adsorbed tartrazine. It is observed that both roughness (Ra = 2.15 μm) and thickness (14.1 μm) increase considerably. This increase is attributed to the presence of adsorbed tartrazine in the MIP.

MIP-PET 2/1 was selected for further adsorption studies and analysis not only for its lower roughness and greater availability of active sites but also for its higher adsorption capacity, as will be seen later.

**Figure 11:**
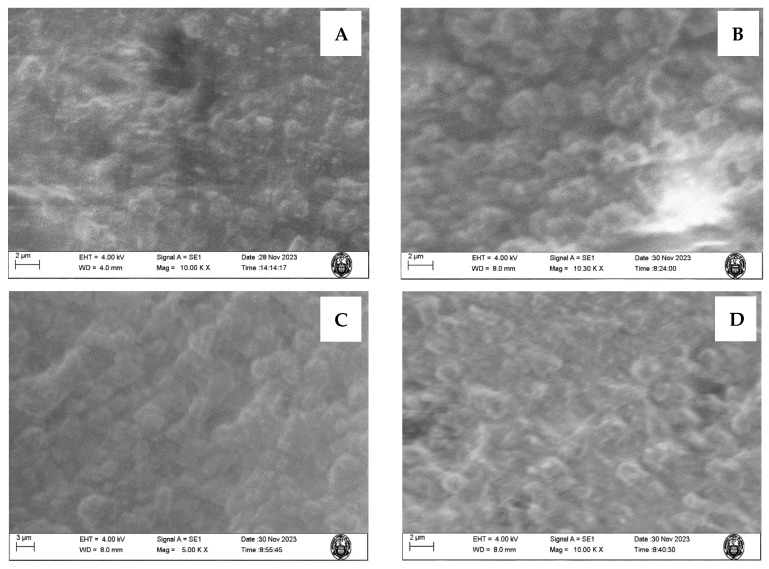
The SEM images of (**A**) MIP–PET con RAFT, (**B**) NIP–PET con RAFT, (**C**) MIP–PET sin RAFT, and (**D**) MIP–PET with tartrazine (MIP–T).

The SEM microscopy of the MIP–PET and NIP-PET treated with RAFT, along with the MIP–PET without RAFT and the MIP-PET with tartrazine, is shown in Figure 11A–D. It can be observed that the MIP-PET C/RAFT exhibits a surface with less roughness compared to the NIP–PET C/RAFT, while the MIP–PET S/RAFT also presents greater roughness. This demonstrates the effect of the RAFT process on obtaining an MIP with a more homogeneous and uniform surface. 

**Figure 12:**
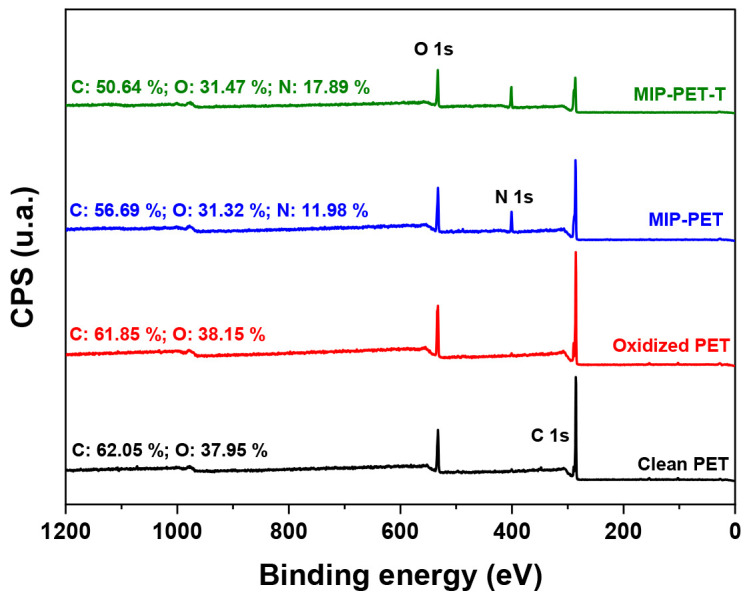
Wide XPS spectrum for clean PET, oxidized PET, MIP–PET, and MIP–PET with tartrazine (MIP–PET–T).

X-ray photoelectron spectroscopy (XPS) is a surface analytical technique that can be used to determine the elemental composition and chemical state of elements present on the surface of the MIP. The overall XPS scans and their elemental compositions for clean PET, oxidized PET, MIP–PET, and MIP–PET with tartrazine are shown in Figure 12.

The spectra of clean PET and oxidized PET showed only carbon (C) and oxygen (O) in their composition. The oxygen content slightly increases in oxidized PET (from 37.95% to 38.15%) due to the conversion of C=O groups to carboxylates (–COOH). In the MIP–PET spectrum, a nitrogen peak appears (11.98%) due to the polymer’s structure, formed by amino units (–NH–) from *N*,*N*′–methylenebisacrylamide. In the case of MIP-PET with tartrazine, the proportion of N increases even further (17.89%) due to the presence of tartrazine inserted into the structure of MIP–PET.

**Figure 13:**
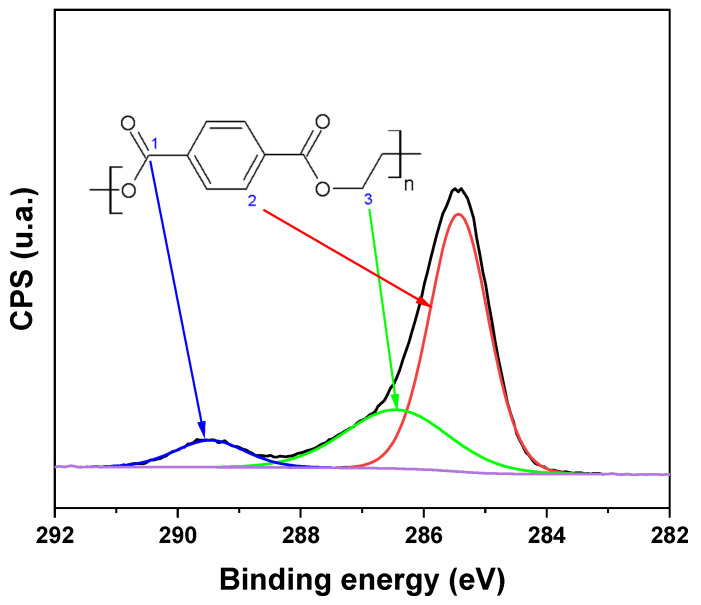
High–resolution spectrum for the C 1s of PET showing the three types of carbon in PET.

Figure 13 shows the high-resolution XPS spectrum for C 1s of PET with the deconvoluted peaks and the chemical structure of PET. PET presents carbon with three different chemical environments, as shown in the expanded XPS spectrum for C 1s. The peak of the aromatic ring’s CC is located at 285.5 eV (carbon type 2). The carbon of the carbonyl (C=O) is shown at approximately 289.5 eV (carbon type 1), and the carbon of the C–O is found at 286.4 eV (carbon type 3). This spectrum will serve as a reference for comparing changes in the carbon’s chemical environment during PET treatment [18,30].

**Figure 14:**
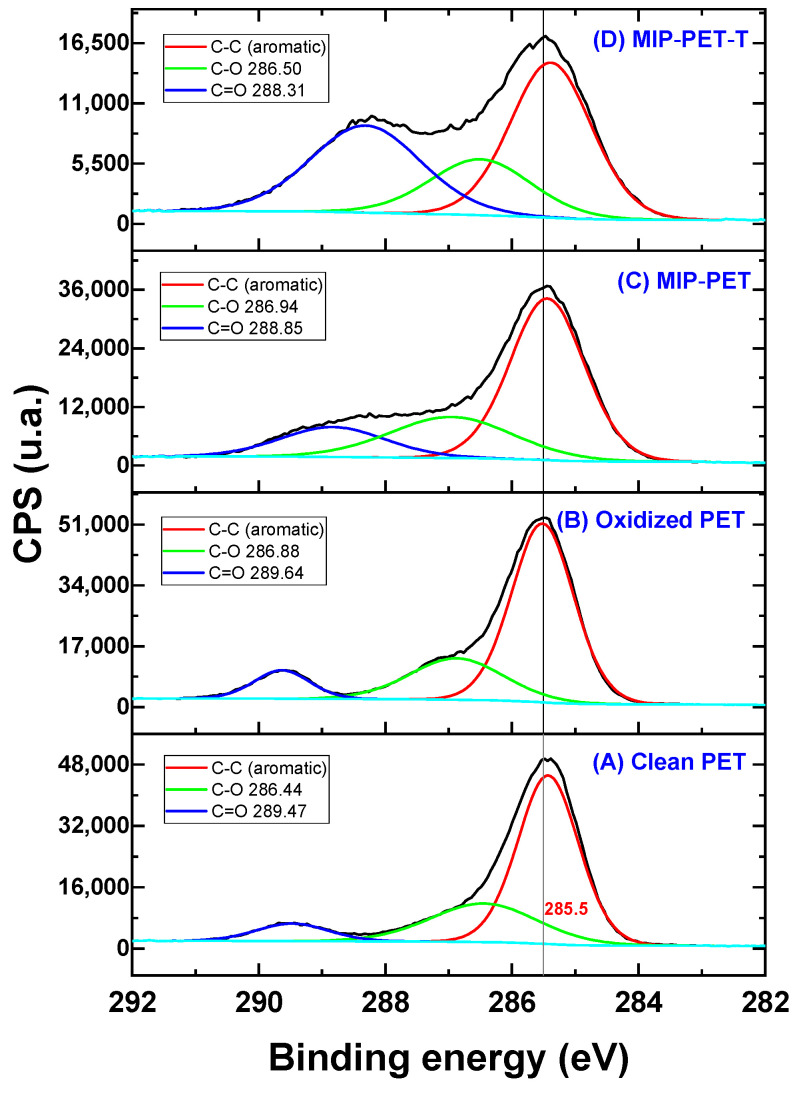
High-resolution spectrum for the C 1s for the different samples: (**A**) clean PET; (**B**) oxidized PET; (**C**) MIP–PET; and (**D**) MIP–PET with tartrazine (MIP–PET–T). The black line corresponds to the original XPS spectrum.

Figure 14 shows the deconvolution of the XPS C 1s spectrum, revealing the carbon components for CC (aromatic), C=O, and O=C–O. The peak of the CC from the aromatic ring remains at 284.8 eV in both the PET plate and the oxidized PET. In the case of MIP-PET, this peak decreases from 67.1% (from oxidized PET) to 59.5% due to the formation of the MIP on the PET. The X-ray photoelectron radiation incident on this material partly penetrates the MIP to reach the PET. In the MIP–PET with tartrazine, the peak of the aromatic CC further decreases to 45.2%, with this percentage mainly corresponding to the aromatic ring of tartrazine.

The peak of O=C–O of the clean PET, located at 286.44 eV, shifts to 286.88 eV in the oxidized PET. This shift is attributed to the conversion of ester groups to carboxylic groups (–COOH), as shown in the reaction in Figure 1. In the case of MIP–PET with tartrazine, the peak shifts to 286.50 eV, and its percentage decreases to 20.3%. This change confirms that the peak corresponds to the carboxylate (–COO–) of tartrazine. Additionally, in this peak, the C–N peak of the crosslinking functional group overlaps [31].

The peak of C=O of the oxidized PET, with an area of 9.5% and a binding energy of 289.64 eV, increases to 24.9% and shifts to 288.83 eV in the MIP–PET. This change is because this last peak corresponds to the C=O of the crosslinker of the MIP. In the case of MIP–PET with tartrazine, the C=O peak shifts even further to 288.31 eV, and its area increases to 34.6%. This change is attributed to the carbonyl group (C=O) of tartrazine, confirming the incorporation of tartrazine into the MIP.

**Figure 15:**
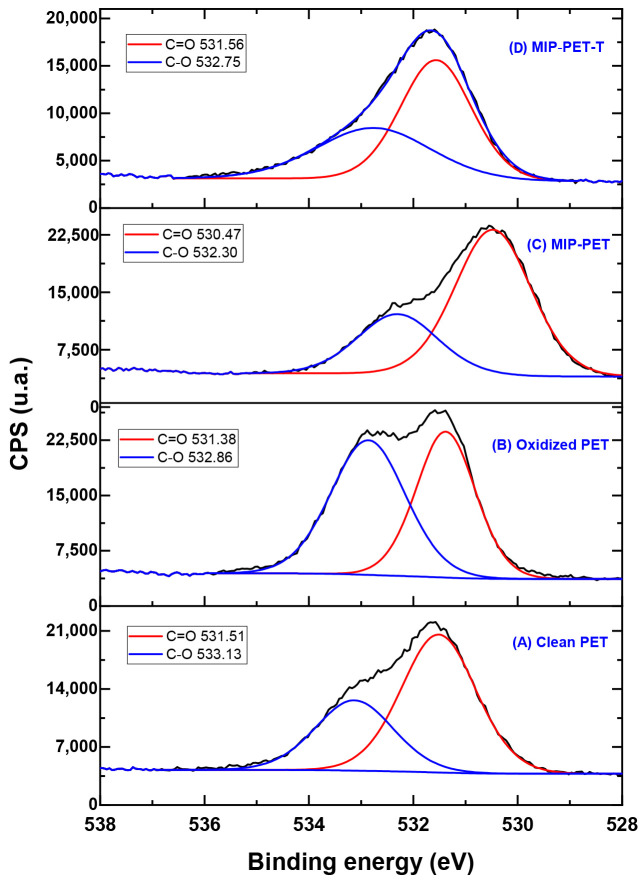
High-resolution spectrum for the O 1s in different samples: (**A**) clean PET; (**B**) oxidized PET; (**C**) MIP–PET; and (**D**) MIP–PET with tartrazine (MIP–PET–T). The black line corresponds to the original XPS spectrum.

Figure 15 displays the deconvolution of the XPS O 1s spectrum, revealing the oxygen components for C=O and C–O (from –COOH) in the different analyzed samples. The intensity of the C–O component peak in clean PET increases from 34.7% to 53.8% in oxidized PET. This increase is due to the oxidation process that occurs through the ester group, as shown in Figure 1. In the case of MIP–PET and MIP–PET with tartrazine, changes in the binding energy and composition of the C–O peak are attributed to the MIP and tartrazine, respectively. Similar explanations can be provided for the changes in the C=O peak for these samples.

**Figure 16:**
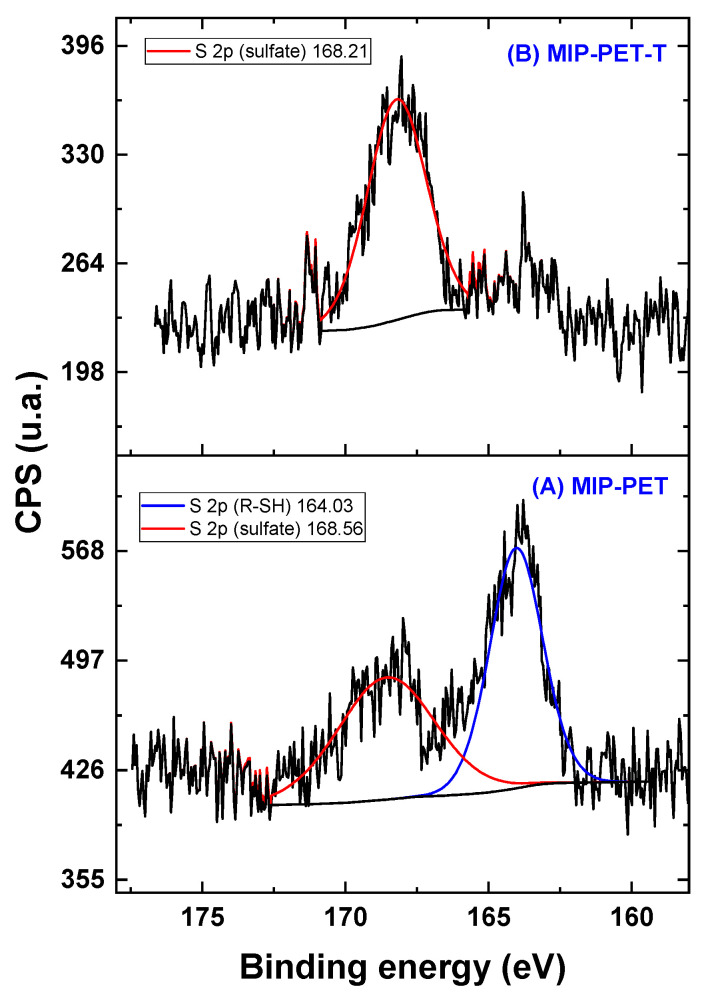
High-resolution spectrum for the S 2p in different samples: (**A**) MIP–PET and (**B**) MIP–PET with tartrazine. The black line corresponds to the original XPS spectrum.

In the total spectrum, the S peaks are not clearly visible; however, the expanded spectrum in Figure 16 reveals the components of S 2p for MIP–PET and MIP–PET with tartrazine (Figure 16). The S 2p spectrum of MIP–PET shows two components: one at 164.03 eV, corresponding to R–SH, and another at 168.56 eV, corresponding to sulfonate. The presence of the R-SH peak confirms that the formation of MIP is carried out using the RAFT reagent, which contains a thiol group. The sulfonate peak is attributed to traces of tartrazine that persist after washing.

In MIP–PET with tartrazine, a peak at 168.21 eV corresponding to the sulfonate of tartrazine is observed. The R–SH peak from the RAFT reagent is not present in MIP–PET with tartrazine due to various treatments that the material undergoes, such as washing.

### 3.4. Adsorption Study

The tests for tartrazine adsorption on the MIP–PET plates were conducted by measuring the remaining amount of tartrazine in the solution using UV–visible spectrophotometry at 427 nm. For this purpose, the MIP–PET plate with the best tartrazine adsorption capacity obtained during the optimization stage was used. This plate was synthesized with a molar ratio of the polymers TZ:AC:NMBA of 1:2:100, a molar ratio of monomers to RAFT reagent of 1330, and a molar ratio of RAFT/KPS of 2:1.

#### 3.4.1. Effect of the pH

**Figure 17:**
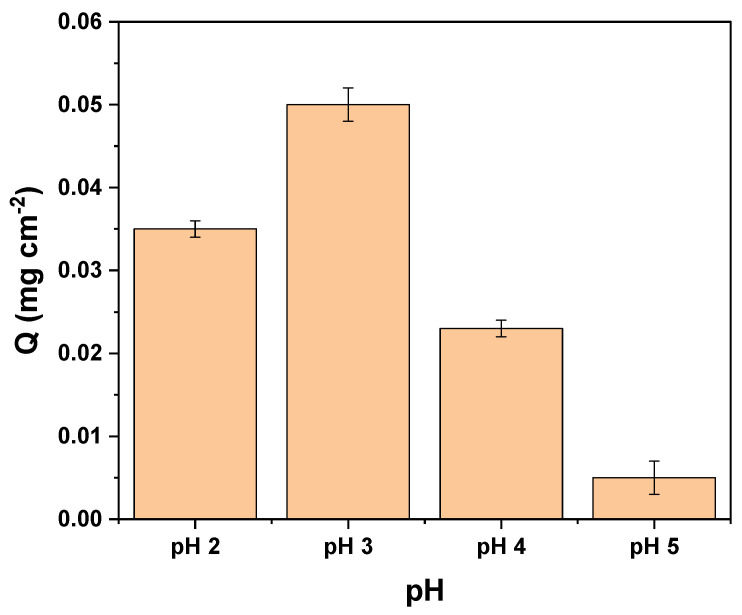
Effect of pH on the adsorption capacity (Q) of MIP–PET. Adsorption of 10 mg L^−1^ tartrazine with agitation time of 60 min.

The effect of the initial pH of the tartrazine solution on the adsorption capacity of MIP–PET plates is shown in Figure 17.

The tartrazine molecule possesses ionizable sulfonate groups (T − SO_3_Na), and the polymeric matrix is formed by amino groups (–NH_2_, pKa = 2.22). At pH 3.0, the amino groups of the polymeric matrix (from the crosslinking monomer) become protonated:

P − NH_2_ + H^+^ ↔ P − NH_3_^+^

T − SO_3_Na → T − SO_3_^−^ + Na^+^

Therefore, the adsorption process at pH 3.0 occurs through electrostatic interaction between the sulfonate and protonated amino groups.

#### 3.4.2. Kinetic Study

**Figure 18:**
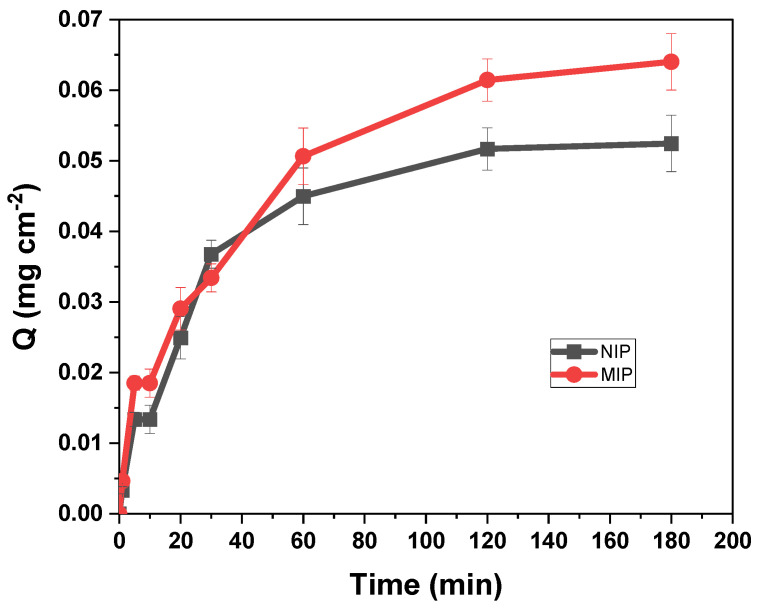
Effect of time on the adsorption of MIP–PET prepared under optimal conditions; 1 cm^2^ plate, [tartrazine] = 10 mg L^−1^, pH 3.

The kinetics of adsorption are important because they can explain the adsorption behavior and establish the contact time for future adsorption tests. The variation of adsorption capacity Q from a 10 mg L^−1^ tartrazine solution at pH 3 as a function of time is shown in Figure 18.

The kinetic curve shows that the adsorption of tartrazine on MIP–PET is a slow process, and equilibrium is reached only after 120 min. This may be due to the limited mobility of tartrazine through the pores.

The pseudo-first-order kinetic model provides information about the rate of occupation of adsorption sites, which is proportional to the unoccupied sites. It was represented by Equation (5) [32]:

(5) $$\log\left(Q_{e}-Q_{t}\right)=logQ_{e}-k_{1}\frac{t}{2.303}$$

where *Q_e_* and *Q_t_* are the amounts of adsorbed tartrazine on the plate (mg cm^−2^) at equilibrium and at time *t* (min), respectively, and *k*_1_ (min^−1^) is the pseudo-first-order rate constant.

The adsorption process can also be described by the pseudo-second-order kinetics, which involves a chemical interaction between the adsorbate molecules and the adsorption sites. It is described by Equation (6) [33]:

(6) $$\frac{t}{Q_{t}}=\frac{1}{k_{2}Q_{e}^{2}}+\frac{t}{Q_{e}}$$

where *k*_2_ (mg cm^2^ min^−1^) is the pseudo-second order kinetic rate constant.

Additionally, kinetic data were evaluated with the intraparticle diffusion model of Morris and Weber by Equation (7) [34]:

(7) $$Q_{t}=k_{id}t^{1/2}+C$$

where *C* is the intercept indicating the thickness of the boundary layer, and *k_id_* is the intraparticle diffusion constant. According to this model, the adsorption process is divided into two stages. The first stage involves the external adsorption of adsorbate molecules on the surface of adsorbent particles. The second stage involves the diffusion of adsorbate molecules within the adsorbent particles.

The corresponding kinetic parameters and the correlation coefficients *R*^2^ of three kinetic models are summarized in Table 2. The high *R*^2^ values and the similarities between *Q_exp_* and *Q_cal_* of the pseudo-first-order model indicate that in the overall process, it fits quite well with the experimental data for the adsorption of tartrazine on MIP–PET.

**Figure 19:**
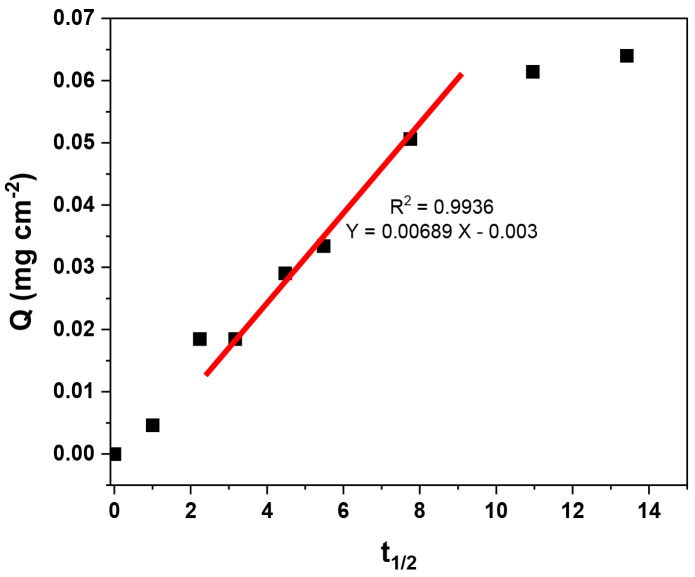
Intraparticle diffusion model of tartrazine in MIP–PET, in the range of 10–60 min.

For a solid–liquid adsorption process, the adsorption kinetics were controlled by three consecutive mass transport steps related to the adsorption of the solute from a solution onto the adsorbent [34]. The steps were (1) film diffusion, (2) intraparticle or pore diffusion, and (3) sorption at interior sites. The third step was rapid and considered insignificant, so the first two steps were the most important. In the range of 10 to 60 min, the adsorption process is controlled by the intraparticle adsorption process without reaching the maximum adsorption capacity because the adsorption process is slow. Above 60 min, the adsorption process involves additional surface phenomena [16] (Figure 19).

#### 3.4.3. Isotherm Study

**Figure 20:**
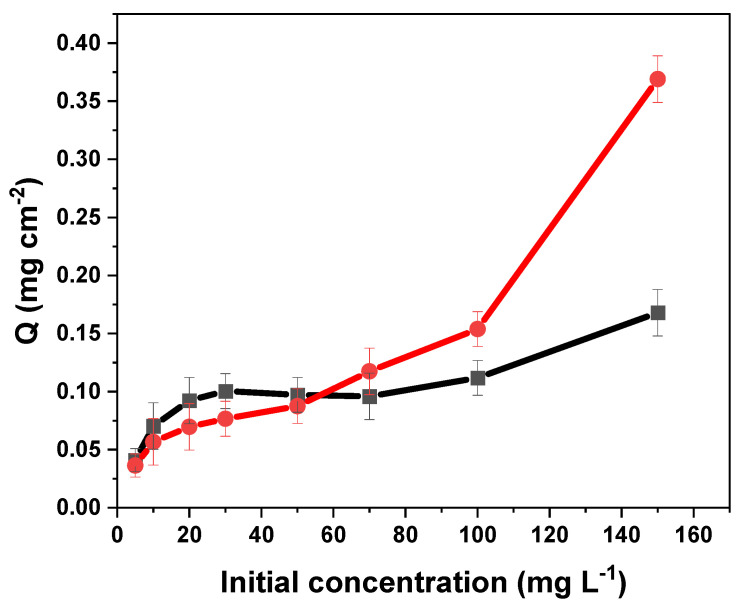
Adsorption isotherm of tartrazine on MIP–PET and NIP–PET. Tartrazine solution at pH = 3 and volume of 10 mL.

The effect of the initial concentration on the adsorption capacity of tartrazine on MIP–PET and NIP–PET are shown in Figure 20. In the initial concentration range of tartrazine from 0 to 60 mg L^−1^, there were no significant differences in the adsorption capacities of MIP–PET and NIP–PET. However, at higher concentrations, MIP–PET showed a higher adsorption capacity compared to NIP–PET.

The adsorption isotherm provides information about the mechanism of the adsorption process. The simplest and yet most useful isotherm, both for physical and chemical adsorption, is the Langmuir isotherm. This model assumes that adsorption is limited to a monolayer, the surface of the adsorbent is homogeneous, the adsorption energy is uniform for all sites, and there is no transmigration of the adsorbate on the surface plane. The Langmuir isotherm is expressed by Equation (8) [35]:

(8) $$\frac{C_{e}}{Q_{e}}=\frac{C_{e}}{Q_{m}}+\frac{1}{bQ_{m}}$$

where *Q_e_* is the adsorption capacity at equilibrium (mg L^−1^), *Q_m_* is the maximum adsorption capacity (mg L^−1^), *b* is the equilibrium constant (L mg^−1^), which is a measure of the adsorbate’s tendency to be adsorbed on the active sites of the adsorbent surface. A high value of *b* represents higher adsorption energy. Although the Langmuir isotherm is the most used binding model for adsorption studies, it cannot be applied to imprinted polymers due to the logarithmic distribution of binding sites in multilayers [36].

The Freundlich isotherm model is an empirical equation and another form of Langmuir that can be applied to multilayer adsorption. This model assumes that the adsorbent surface is heterogeneous and the active sites and their energies are exponentially distributed. The Freundlich isotherm is expressed by Equation (9) [36]:

(9) $$logQ_{e}=\frac{1}{n}logC_{e}+\log K_{f}$$

where *K_f_* (mg g^−1^) is the adsorption coefficient and represents the adhesion ability of the adsorbate to the adsorbent. The term *1*/*n* indicates the adsorption intensity of the adsorbate to the adsorbent or heterogeneous surface. If the slope *1*/*n* lies between 0 and 1, it indicates a favorable adsorption isotherm. When this value is closer to zero, the adsorbent surface is more heterogeneous.

The constants and correlation coefficients of the Langmuir and Freundlich models are shown in Table 3.

The isotherms fit a Freundlich model in the range of 0 to 100 mg L^−1^. The Freundlich isotherm allows for a better analysis of the different binding sites existing in the MIP compared to the Langmuir model.

The obtained value of *n* (3.66) indicated that the adsorption of tartrazine is favorable, but being greater than 1, it also indicates that the system is heterogeneous. This value of *n* can be an indicative parameter of the effect of RAFT polymerization on the homogeneity of the MIP [36]. Specifically, with RAFT polymerization, a smaller value of *n*, close to 1, would be expected compared to the MIP formed without the RAFT effect. This would indicate greater homogeneity of the MIP with RAFT.

#### 3.4.4. Selectivity Study

Selectivity is an important property for assessing the ability of the MIP as a preferential adsorbent for the analyte. To evaluate the selectivity of the MIP and NIP, the molecular imprinting factor (MIF) and Selectivity Factor (SF) were determined, which are calculated according to Equations (10) and (11) [37]:

(10) $$\mathrm{Imprinting}\;\mathrm{Factor}\;\left(\alpha \right)=\frac{\%\;Removal\;\left(MIP\right)}{\%\;Removal\;\left(NIP\right)}$$

(11) $$\mathrm{Selectivity}\;\mathrm{Factor}\;\left(\beta \right)=\frac{\alpha \;\left(tartrazine\right)}{\alpha \;\left(interferent\right)}$$

**Figure 21:**
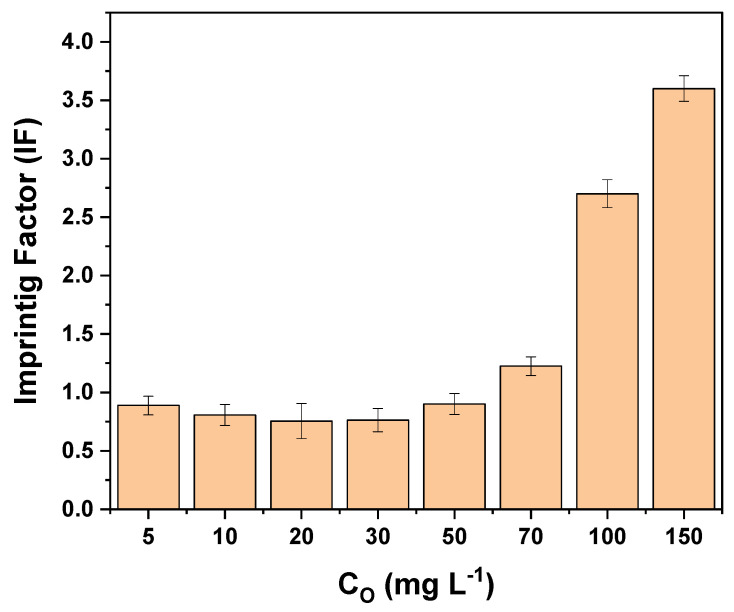
Imprinting factor (IF) of MIP–PET according to the initial concentration of tartrazine. Adsorption with 1 cm^2^ plate, 2-h agitation, 10 mL of tartrazine solution, and pH 3.

The molecular imprinting factor of tartrazine according to the initial concentration is shown in Figure 21. It can be observed that up to 50 mg L^−1^, the MIF is not favorable and is less than 1, while from 70 mg L^−1^ onwards, it is greater than 1. A concentration of 100 mg L^−1^ was chosen for the selectivity tests.

The effects of 4 dyes were studied: Basic Red 46, Methyl Green, Sunset Yellow, and Yellow HE3G. The amount of dye adsorbed by the MIP–PET and NIP–PET was measured using their UV–visible spectrum at the maximum wavelength of each dye. The values of the imprinting factor (IF) and selectivity factor (α) are shown in Table 4.

The selectivity of the MIP is related to the affinity of the functional groups, as well as the size and shape of the templates and recognition cavities. Since tartrazine is a relatively large molecule, it does not form cavities with a high degree of molecular recognition, explaining the low imprinting factor at low concentrations. However, at higher concentrations and considering the greater heterogeneity of the MIP, adsorption by the MIP becomes predominant compared to the NIP.

One of the most common dyes found alongside tartrazine in foods is sunset yellow, as they both have a similar structure. Both compounds are yellow in aqueous solutions and contain azo and sulfonate groups. Due to these characteristics, sunset yellow exhibits a lower selectivity factor compared to the studied dyes. In previous studies on the selectivity of MIP synthesized by the precipitation method (without RAFT agent), sunset yellow presented a selectivity factor of 1.02. In the present work, this factor increases to 2.26, indicating an improvement in MIP synthesis due to the RAFT polymerization effect.

#### 3.4.5. Reusability Study

**Figure 22:**
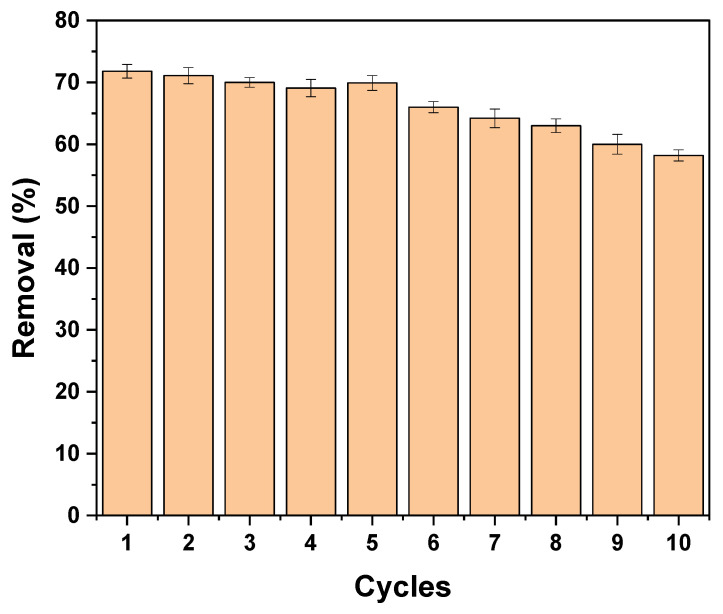
Performance of the MIP-PET after each use over 10 cycles. Concentration of 10 mg L^−1^ tartrazine, 10 mL at pH 3.

The reusability of MIP–PET is an important parameter for its use in multiple applications. The potential for reusability of MIP–PET over 10 identical uses is shown in Figure 22. As observed in the figure, there is no significant decrease until the fifth use. After this point, the removal percentage decreases significantly. These results indicate that MIP–PET can be used up to five times without significantly losing its tartrazine removal capacity.

### 3.5. Digital Image Colorimetry Protocol

#### 3.5.1. Smartphone Image Capture

**Figure 23:**
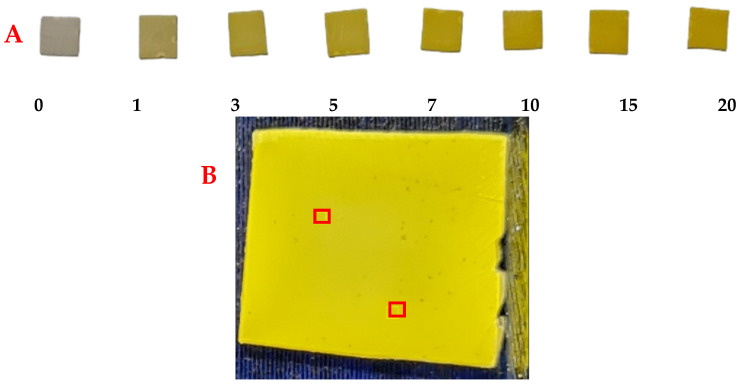
MIP–PET plates exposed to tartrazine solutions for digital image colorimetry with a smartphone. (**A**) Plates exposed to different concentrations of tartrazine (mg L^−1^) on the MIP–PET and (**B**) image of MIP-PET plate with a selected area of 16 × 16 pixels. Adsorption conditions: 1 cm^2^ plate, 3 mL of tartrazine at pH 3, agitation time 2 h.

The adsorption tests were conducted using MIP–PET plates with tartrazine solutions ranging from 0 to 20 mg L^−1^ and with a reduced volume compared to previous tests (3 mL). This volume reduction was performed to ensure that the MIP–PET adsorbed all the tartrazine, allowing the color of the plate to be proportional to the amount of tartrazine present in the solution (Figure 23A). The pH was maintained at 3.0, with an adsorption time of 2 h and a plate size of 1 cm^2^.

Once the image of the plates was captured, using ImageJ (1.53k 6 July 2021) software, two regions of 16 × 16 pixels were selected from each image, and the RGB values were recorded (Figure 23B). These values, along with the equations mentioned earlier (Equations (3) and (4)), were converted to CMYK and HSV. RGB values represent the intensity of the primary colors: red, green, and blue. To relate them to other colors, CMYK channels (cyan, magenta, yellow, and black) are used, representing the amount of each of these colors in an image. On the other hand, HSV values represent the hue (color), saturation (color intensity), and value (color brightness) in a different color system.

**Figure 24:**
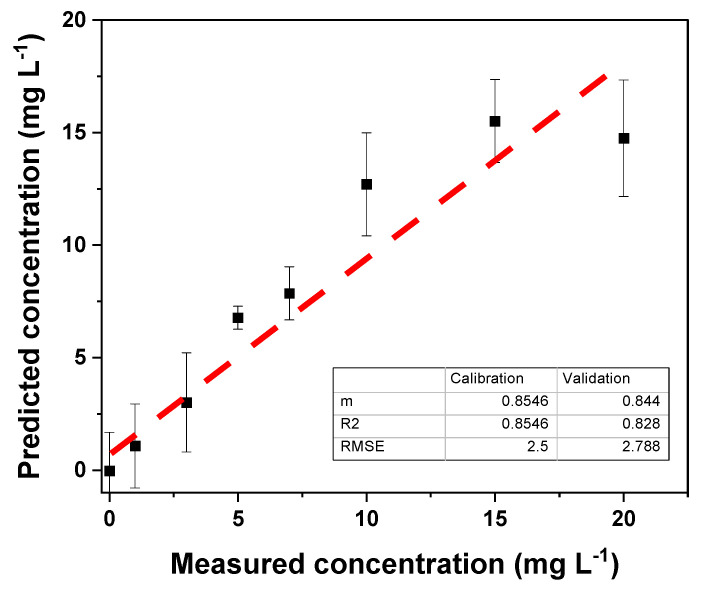
Results of the PLS calibration in the concentration range of 0–20 mg L^−1^.

Partial least squares regression (PLS) was performed on the RGB, CMYK, and HSV color values using The Unscrambler X 10.1 software. This multivariate calibration involves a variable reduction to principal components (or factors) and subsequent multiple linear regression with the selected principal components. To evaluate the working range of the PLS regression, cross-validation, the *R*^2^ value between the measured and predicted values, and the root mean square error (RMSE) were used. Reduction to four principal components (PCs) extracted the following regression in the tartrazine concentration range from 0 to 20 mg L^−1^ (Figure 24).

**Figure 25:**
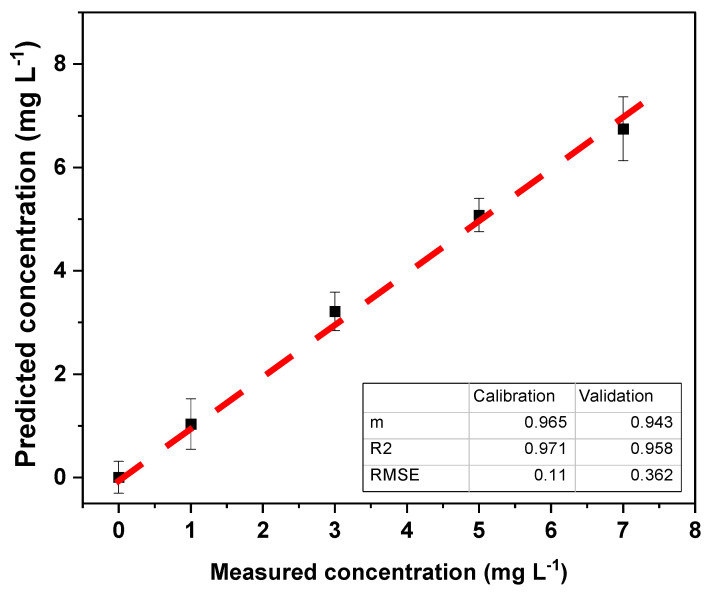
Results of the PLS calibration in the concentration range of 0–7 mg L^−1^.

Since the *R*^2^ and m values were not optimal (close to 1), the range of 0 to 7 mg L^−1^ was selected to assess the working range (Figure 25). In this range, an increase in the *R*^2^ values and slope was observed, while the RMSE decreased, indicating an improvement in the PLS model for the tartrazine concentration range of 1.0 to 7.0 mg L^−1^.

**Figure 26:**
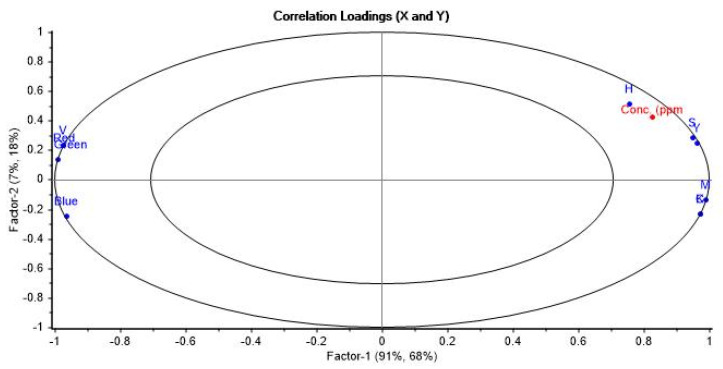
Biplot graph showing the correlation between the RGB, CMYK, and HSV color variables with the predicted concentration of tartrazine in the range of 0–7 mg L^−1^ using 4 principal components.

Considering this working range from 1.0 to 7.0 mg L^−1^, LOQ was 1.0 mg L^−1^, and the LOD was 0.3 mg L^−1^. Upon closer examination of the principal component analysis (PCA) performed in the PLS calibration, a biplot graph (Figure 26) allows for visualization of the relationship between the original variables and the measured tartrazine concentration.

The biplot reveals that the concentration of tartrazine positively correlates with the H (hue), S (saturation), and Y (yellow) channels, which define the type of yellow color, its intensity, and the yellow color itself, respectively. The concentration is inversely proportional to the V (brightness) value, indicating that higher yellow intensity corresponds to lower brightness. There is also an inverse correlation with the R (red) and G (green) values, the primary colors whose mixture yields yellow.

The optimized regression model was used to evaluate the model with repeatability tests of 10 samples of 5 mg L^−1^, and the results obtained are shown in Table 5:

The repeatability of the method at the 5 mg L^−1^ level was found to be +0.74 mg L^−1^ with a relative standard deviation of 14.9%. This was a relatively high value, but it should be considered that it is a practical method that uses no expensive equipment and is easy to use. On the other hand, the accuracy of the proposed method was 99.4%.

#### 3.5.2. Analysis of Carbonated Beverages

The application for determining tartrazine using MIP–PET was carried out on samples of local carbonated beverages. Two degassed carbonated beverages were analyzed to determine the concentration of tartrazine and compared with the results obtained by spectrophotometric analysis—standard addition. The results obtained are shown in Table 6, and no significant differences were observed between the proposed method and the UV–visible reference method.

This evidences that the proposed method is a suitable alternative for determining tartrazine in carbonated beverages rapidly, accurately, and simply.

## 4. Conclusions

This study successfully developed a molecularly imprinted polymer (MIP) platform on polyethylene terephthalate (PET) for the determination of tartrazine in carbonated beverages using a smartphone. The MIP–PET platform was prepared via RAFT polymerization, yielding a material with high adsorption capacity (0.057 mg cm^−2^) and enhanced selectivity.

Optimal conditions for MIP–PET preparation were determined, including the molar ratio between tartrazine, monomers, and the RAFT agent. The MIP-PET was characterized by FTIR, Raman spectroscopy, XPS, and confocal microscopy, confirming the formation of the MIP layer on PET (film thickness of 7.0 µm).

Kinetic and isotherm studies revealed that the adsorption process of tartrazine on MIP-PET follows a pseudo-first-order model and conforms to a Freundlich model. A method for quantifying tartrazine in carbonated beverages using MIP–PET and a smartphone was developed.

The method developed for quantifying tartrazine in carbonated beverages using multivariate PLS regression showed a working range from 1.0 to 7.0 mg L^−1^, with a detection limit of 0.3 mg L^−1^. The method’s repeatability was 4.97 mg L^−1^ (+0.74) for 10 independent repetitions of 5 mg L^−1^. The concentration of tartrazine in two local carbonated beverages (14.1 and 16.5 mg L^−1^) was determined, yielding results comparable to the UV–visible spectrophotometric method.

The results of this study suggest that the developed MIP–PET is a promising tool for the detection and quantification of tartrazine in carbonated beverages and other food matrices. It is easy to use, cost-effective, accurate, precise, and efficient. Furthermore, it sets a precedent for enhancing analysis with digital images using artificial intelligence and machine learning tools.

## Figures and Tables

**Table 1:** Values of Q obtained according to the ratio of MF + ME/RAFT. MIP-PET plate of 1 cm × 1 cm; 10 mL of 10 mg L^−1^ tartrazine at pH 3 (*n* = 3).

MIP-PET	Ratio (MF + ME)/RAFT	Q (mg cm^−2^)
MIP1	1330	0.029 ± 0.002
MIP2	560	0.025 ± 0.001
MIP3	280	0.023 ± 0.003

**Table 2:** Parameters of the different kinetic models for the adsorption of tartrazine on the MIP–PET plates.

Kinetic Model	Parameters			
Pseudo-first order	*k*_1_ = 0.023	*Q_cal_* = 0.059	*Q_exp_* = 0.064	*R*^2^ = 0.9977
Pseudo-second order	*k*_2_ = 0.013	*Q_cal_* = 0.00373	*Q_exp_* = 0.064	*R*^2^ = 0.9759
Intraparticle Diffusion	*k*_1_ = 5.046			*R*^2^ = 0.9593

**Table 3:** Constants and correlation coefficients of the adsorption isotherm models for tartrazine on MIP–PET.

Adsorption Model	Parameters		
Langmuir	*Q_m_* = 0.1532	*b* = 0.0785	*R*^2^ = 0.9006
Freundlich	*n* = 3.66	*K_f_* = 0.0375	*R*^2^ = 0.9354

**Table 4:** Selectivity parameters of the MIP compared to the NIP for different dyes (100 mg L^−1^, pH 3.0).

Dye	Imprinting Factor (IF)	Selectivity Factor (β)
Basic red 46	0.88	3.08
Methyl green	0.80	3.39
Sunset yellow	1.20	2.26
Yellow HE3G	0.40	6.78
Tartrazine	2.70	---

**Table 5:** Values of repeatability for 10 independent samples.

Sample	Measured Value (mg L^−1^)
M1	4.14
M2	4.51
M3	6.56
M4	4.44
M5	5.62
M6	5.01
M7	4.15
M8	5.36
M9	5.02
M10	4.89
Average	4.97
Stand. Desv.	0.74

**Table 6:** Comparative results of tartrazine in two samples of carbonated beverages.

Sample	UV–Vis Method	Proposed Method (Smartphone)
M1	13.6 ± 0.1 (*n* = 3)	14.1 ± 0.3 (*n* = 3)
M2	16.8 ± 0.2 (*n* = 3)	16.5 ± 0.2 (*n* = 3)

## Data Availability

Data are contained within the article.
